# A Case Report of Acute Esotropia in a Young Woman following Heroin Withdrawal

**DOI:** 10.1155/2015/740710

**Published:** 2015-05-17

**Authors:** Bethel Shiferaw, Ebisa Bekele, Sara Syed, Lu Fan, Nirav Patel, Samia Qazi, Nicolas Biro

**Affiliations:** ^1^Department of Internal Medicine, Nassau University Medical Center, 2201 Hempstead Turnpike, East Meadow, NY 11554, USA; ^2^Department of Ophthalmology, Nassau University Medical Center, 2201 Hempstead Turnpike, East Meadow, NY 11554, USA

## Abstract

*Introduction*. Esotropia is a form of strabismus that can give the affected individual a “cross-eyed” appearance. Acute onset of esotropia is an uncommon form; in the vast majority of cases, no underlying neurological etiology is found. *Case Presentation*. A 22-year-old female with a long history of opiate abuse presented with acute onset of diplopia. She noted her eyes were crossing and started seeing double. She stopped using heroin 11 days prior to presentation. There was large inward deviation of her left eye. Convergence was difficult and accompanied by horizontal nystagmus. Diplopia resolved by covering each eye. Further investigations including imaging studies were normal. *Discussion*. Acute onset esotropia is rare and must be investigated right away to exclude central nervous system pathologies, where no opiates use is reported. Diplopia in the form of acute esotropia may manifest in up to 30% of individuals undergoing heroin withdrawal. Evaluating acute esotropia requires detailed information of medical history with an emphasis on drug use. *Conclusion*. Acute onset esotropia with double vision can be caused by abrupt withdrawal of opiates. This case should serve to raise awareness among health care professionals, to avoid costly and unnecessary diagnostic evaluations and interventions.

## 1. Introduction

Esotropia is a form of strabismus or “squint” in which one or both eyes turn inward. The condition can be present constantly or occur intermittently and can give the affected individual a “cross-eyed” appearance.

Esotropia can be caused by structural restriction of eye movements (e.g., thyroid eye disease, fracture), palsies (6th nerve, increased intracranial pressure), skull base tumour or lesion (e.g., Arnold Chiari malformation), autoimmune disease (e.g., myasthenia gravis) or idiopathic. People suffering from this condition might complain of double vision (diplopia). Acute onset of esotropia in the presence of full range of ocular movements is an uncommon form; in the vast majority of cases, no underlying neurological etiology is found [1].

While double vision is a known symptom of heroin withdrawal, nearly all patients complain of some impairment of vision [2]. The first cases of diplopia or blurred vision following heroin withdrawal were reported in returning US soldiers who served in Vietnam with an incidence of 10%–33.3% [3].

Due to the current resurgence of heroin use, we present case of a young woman who presented to our hospital with acute onset double vision during the beginning of her heroin detoxification.

## 2. Case Presentation

A 22-year-old Caucasian female with a long history of opiate abuse was admitted from the neurology clinic for acute onset of diplopia. Patient stated that, upon awakening 4 days prior to presentation, she noted her eyes were crossing and started seeing double. Her symptoms persisted for several days and she presented to the hospital.

Our patient began using prescription narcotics recreationally with increased frequency. As costs grew, she switched to snorting heroin. She progressed to an average use of 10–15 bags of heroin per day. She stopped using narcotics 11 days prior to presentation. The patient denied pain with eye movement, although she did feel eye strain. She denied headaches, photophobia, fever/chills, rashes, myalgia, nausea, vomiting, abdominal cramping, or diarrhea. There is no recent infection, trauma, sick contacts, or travel history. Pt had motor vehicle accident 4 years priorly without neurological sequelae. The patient denied taking prescription medications currently or smoking tobacco products but admits to drinking alcohol occasionally.

Physical exam included the following: vital signs were BP: 107/73, P: 91, T: 98.5, and RR: 20. There was large inward deviation of her left eye, measuring >40 prism diopters of esotropia. Fine horizontal nystagmus on abduction was noted in each eye. Convergence was difficult and accompanied by horizontal nystagmus. Motility of extraocular muscles was intact. Diplopia resolved by covering each eye. The rest of the medical and neurological examination was within normal limits.

Investigation was as follows: WBC was 7.32, hemoglobin was 14.8, and platelets were 268. Organ function tests were within normal range. Lyme, TSH, RPR, ANA, anti-DNA, and RF were negative. Urine toxicology including opiates and PCP was negative. CT head identified no intracranial abnormality. MRI and MRA of the brain were normal.

The patient's double vision throughout her admission improved slightly. A lumbar puncture was deferred as patient showed improvement after admission and showed no neurological signs. Patient was discharged home with ophthalmology follow-up. At each follow-up visit, her deviation and diplopia continued to improve. Two months after presentation, she was able to see single intermittently. At her last visit, around 10 weeks since her symptoms began, her diplopia and esotropia have completely resolved.

## 3. Discussion

Unlike common forms of esotropia, which presents in childhood or develops slowly in adults, acute onset esotropia is rare. Therefore it must beinvestigated right away to exclude possible pathologies mentioned above, where no opiates use is reported. Investigationincludes checking motility, measuring deviation, looking for papilledema and ptosis, and performing a full neurological exam.

Various hypotheses were proposed for development of acute onset of esotropia in adults with heroin withdrawal [4]. Opioid use causes miosis, which produces an increased depth of focus and a decreased need to accommodate. Heroin withdrawal has a dramatic impact on the human central nervous system's function affecting the balance between the oculomotor system and the sensory mechanisms of binocular vision [5]. The “sudden parasympatholytic state with pupillary dilation and paralysis of the ciliary muscle” associated with heroin withdrawal may precipitate decompensation of fusion, leading to esotropia [5–7].

Diplopia in the form of acute esotropia may manifest in up to 30% of individuals undergoing heroin withdrawal [6]. Another prospective study in 2004 on patients attending a 5-day opiate detoxification program indicated that 72.5% of them had diplopia and/or blurred vision, which is higher than previously reported [8].

Heroin withdrawal signs and symptoms may begin 6 to 12 hours after the last dose, typically peaking within 24 to 48 hours of onset, but may persist for several days. Our patient started having the symptoms towards the end of first week after cessation of using heroin. This might highlight that acute esotropia after heroin withdrawal might present at a later time than the usual heroin withdrawal sign and symptoms.

When evaluating cases of acute esotropia, a detailed medical history with an emphasis on drug use plays an important role [4, 6, 9]. This approach will help to avoid costly and unnecessary diagnostic evaluations and interventions.

There is no known treatment to reverse or decrease symptoms, but eye patching or prismatic correction can provide symptomatic relief. Resolution of signs and symptoms usually begins 1 to 2 months after onset [6]. In the longer term, botulinum toxin to the medial rectus muscle or surgical intervention may be undertaken if the deviation persists [10]. Our patient's double vision continued to improve slowly and the patient continued to occlude one eye to give relief from the diplopia; therefore reassurance was given along with monitoring.

## 4. Conclusion

Acute onset esotropia with double vision can be caused by abrupt withdrawal of opiates, which can be frustrating to patients. We report a case of acute esotropia in a young woman for which the cause is most likely heroin withdrawal. This case presentation should serve to raise awareness among health care professionals for timely diagnosis of this condition and help reduce unwarranted costly imaging as well as providing reassurance to patients.
